# Genetic characterization of hull color using BSR-Seq and genome re-sequencing approaches in foxtail millet

**DOI:** 10.3389/fpls.2022.1019496

**Published:** 2022-10-03

**Authors:** Bohong Tian, Lixin Zhang, Jinghuang Hu, Yanli Liu, Lulu Zhou, Wenchao Ping, Jingwei Zou, Hongjie Li

**Affiliations:** ^1^ Cangzhou Academy of Agricultural and Forestry Sciences, Cangzhou, China; ^2^ The National Engineering Laboratory of Crop Molecular Breeding, Institute of Crop Sciences, Chinese Academy of Agricultural Sciences, Beijing, China

**Keywords:** *Setaria italica*, hull color, BSR-Seq, genome re-sequencing, molecular mapping

## Abstract

Hull color of foxtail millet is an important indicator of certain nutritional quality parameters. An F_2:6_ recombinant inbred line (RIL) population developed by crossing a yellow-hulled cultivar Yugu 5 and a brown-hulled cultivar Jigu 31 was used to determine the genetic control of the hull color trait. This population segregated for yellow and brown hull colors in a ratio of 2:1, indicating that hull color is regulated by multiple genetic loci. A bulk segregant analysis-RNA sequencing (BSR-Seq) approach performed using the RNA bulks from 30 lines with brown and yellow hull colors each identified three genomic regions on chromosomes 1 (4,570,517-10,698,955 bp), 2 (40,301,380-46,168,003 bp), and 3 (44,469,860-50,532,757 bp). A new QTL for brown hull color of Jigu 31, *QHC.czas1*, was detected between bin markers Block43 and Block697 on chromosome 1 with the genetic linkage map constructed by re-sequencing a subset of the 147 RILs. This QTL explained a high level of phenotypic variation ranging from 28.0% to 47.0%. The corresponding genomic region of this QTL in the foxtail millet reference genome overlapped with that detected on chromosome 1 by the BSR-Seq analysis. Nineteen genes associated with biosynthesis of anthocyanin were annotated in this genomic region. Gene *Si1g06530* encoding a SANT/Myb domain protein was highly expressed in developing panicles and seeds, which warrants further verification as the candidate gene for the brown color hull of Jigu 31. Moreover, several annotated genes for biosynthesis of anthocyanin were identified in the genomic regions of chromosomes 2 and 3.

## Introduction

Foxtail millet [*Setaria italica* (L.) P. Beauv.] has been grown as a staple food crop for several thousands of years in China. It is also planted in other parts of the world such as eastern Asia, north and south Americas, Africa, and Europe (Yang et al., 2012; Hermuth et al., 2016; Singh et al., 2017). This cereal crop is considered a new model species for functional genomics studies of C4 plants (Peng and Zhang, 2021). The unique characteristics of foxtail millet, for instance, small genome (~515 Mb), short stature and growth duration, self-pollination, and abiotic stress tolerance, have attracted extensive attentions, despite remarkable decline in its production due to the limitation of market demand (Panchal et al., 2022). Foxtail millet is known for its human-friendly nutritional properties, such as dietary fiber, antioxidants, phytochemicals and polyphenols (Muthamilarasan et al., 2016; Sharma and Niranjan, 2018; Yousaf et al., 2021).

Foxtail millet comprises a layer of husk or hull out of seeds (Sharma and Niranjan, 2018). Pigmentation occurs in this layer, making colorful appearance in hulls of millet seeds. Hull colors of foxtail millet were associated with certain nutritional qualities, such as contents of protein, lysine, and fat (He et al., 2002). The correlation between hull color and selenium content was reported (Liu et al., 2009). Hull color also was believed to have a relationship with the magnitude of bird-damage on this millet crop at the maturity stage (Xia et al., 2014). Therefore, hull color is regarded as a useful phenotypic indicator for indirect selection of quality and other agronomic traits in foxtail millet breeding.

Foxtail millet has evolved different hull colors. The majority of the 878 (77.46%) accessions of the Chinese core collections are yellow hull-colored genotype, in addition to small proportions of genotypes with red (8.27%), white (5.64%, and orange (4.20%) hull colors (Wang et al., 2016). Multiple loci regulating different hull colors have been studied using various methods. An early study localized genes for red and green hull colors on chromosomes 1 and 2, respectively, using a series of trisomic lines of foxtail millet (Gao et al., 2003). The foxtail millet anuploids are able to assign target genes on particular chromosomes. Molecular markers are promising tags of genes conferring traits of interest when linkage relationships are established. Genes governing agronomic traits can be localized on specific chromosomal regions as most gene-linked molecular markers possess unique locations on chromosomes (Diao, 2005).

The release of the genomic sequences of foxtail millet greatly facilitates dissection of genes conferring agronomic traits (Bennetzen et al., 2012; Zhang et al., 2012). The popularly used types of molecular markers shifts from sporadically distributed markers such as simple sequence repeat (SSR) to enormous single nucleotide polymorphism (SNP)-based markers throughout the genome. High-density genetic maps can be constructed with SNP markers, which increase the precision of gene localization. A quantitative trait locus (QTL), *Sihc1*, for green hull color was located in a 354.84 kb physical region using the SNP markers generated by a restriction site-associated DNA sequencing (RAD-seq) analysis (Wang et al., 2017). That study predicted the candidate genes for the green hull color. A genome-wide association study (GWAS) project identified several hull color-associated SNP variants on four chromosomes (Jia et al., 2013).

Cultivars Jigu 31 and Yugu 5 differ in their hull colors. This study was carried out to identify loci conferring hull color using a recombinant inbred line (RIL) population of cross Jigu 31 × Yugu 5 with the aid of SNP markers generated by bulked segregant analysis-RNA-sequencing (BSR-Seq) and genome-re-sequencing methods.

## Materials and methods

### Plant materials and phenotyping

An F_2:6_ RIL population consisting of 283 lines was developed from consecutively self-crossing progenies of the cross between Yugu 5, a yellow hull colored cultivar, and Jigu 31 with a brown hull color. This RIL population and their parents were grown in Cangzhou, Hebei province (33°13’N, 116°47’E) in summer and Sanya, Hainan province (18°35’N, 109°19’E) in winter in 2016 and 2017. All plant materials were planted in a randomized complete block design with two replicates. Each plot consisted of single row 5 m long, 74 cm spacing between rows, and about 7 cm between plants. Three panicles were randomly harvested from each line, hand-threshed, and visually observed for their hull colors. A Chi-squared (χ^2^) was performed with SAS version 9.3 (SAS Institute Inc., Cary, NC, USA) to examine the goodness of fit for the observed separation of yellow and brown hulled RILs from the expected separation ratio for single gene inheritance (1:1) or for multiple gene inheritance (2:1 or others) (Sandler, 2000).

### BSR-seq analysis

Thirty lines with consistent brown or yellow hull colors across different experimental locations were separately selected to form the bulked samples (Bulk-Y and Bulk-B) for BSR-Seq analysis. Total RNA was extracted from leaf segments using the RNAsimple Total RNA Kit (Tiangen, Beijing, China) for constructing the RNA libraries. Raw sequencing read generated by RNA-Seq in a platform of Illumina HiSeq4000 were quality controlled using Trimmomatic v0.36 software (Bolger et al., 2014). Clean reads were aligned to the *Setaria italica* reference genome (http://plants.ensembl.org/Setaria_italica/Info/Annotation/#assembly) (Dobin et al., 2013). The alignment of uniquely mapped reads was masked for PCR duplications prior to SNP and InDel calling with small variant caller Strelka v2 (Kim et al., 2018). The high quality SNPs and InDels with sequencing depth >6 were used to detect the trait-associated variants through BSA with the criteria of allele frequency difference (AFD) >0.8 and *P-*value of the Fisher’s exact test on read count data <1e-10 (Xie et al., 2020).

### Construction of genetic linkage map

A genetic linkage map was constructed by re-sequencing 147 RILs randomly selected from the population of the cross Yugu 5 × Jigu 31 (Tian et al., 2021). Briefly, genomic DNA libraries prepared from leaves of each line and their parents were sequenced on the Illumina Hi Seq2500 (Illumina, Inc., San Diego, CA, USA). The high quality reads were aligned to the *S. italica* reference genome *via* the Burrows-Wheeler aligner (Li et al., 2009). After the realignment and base recalibration with the Genome Analysis Toolkit (GATK) v3.6 (McKenna et al., 2010), the data set was subjected to SNP calling using GATK and SAMtools (Li and Durbin, 2009). SNP loci polymorphic between the parents were subjected to bin calling. A linkage map was constructed based on the recombination bins using HighMap software (Liu et al., 2014). The composite interval mapping (CIM) method in Windows QTL Cartographer 2.5 was used for QTL calling with the logarithm of odds (LOD) threshold of 3.0 (Wang et al., 2010). The percentage of the phenotypic variance explained by a QTL was indicated by the determination coefficient (*R* ^2^%).

### Analysis of candidate genes in the target mapping intervals

The QTL for hull color were physically mapped by aligning the sequences of the QTL-flanking markers against the reference genome of foxtail millet with Phytozome *Setaria italica* v2.2 (https://phytozome.jgi.doe.gov/pz/portal.html#!info?alias=Org_Sitalica). Genes in the target genomic intervals were annotated and those related to anthocyanin synthesis were further analyzed for their expression by MDSi: Multi-omics Database for *Setaria italica* (http://foxtail-millet.biocloud.net/home) (Zhang et al., 2021).

## Results

### Phenotypes of hull color

**Figure 1:**
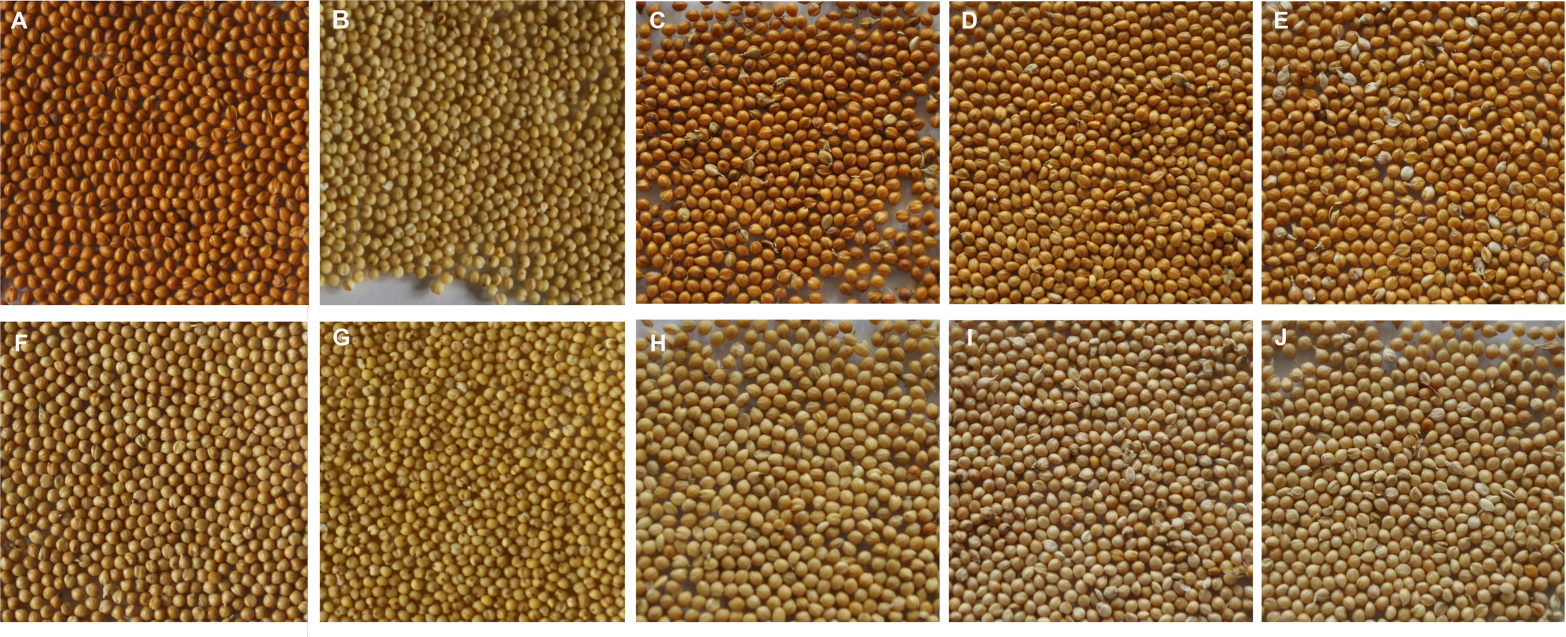
Phenotypic performances of hull and grain colors of foxtail millet cultivars Yugu 5 and Jigu 31 and the selected lines from the cross Yugu 5 × Jigu 31. **(A, B)**, brown hull color and yellow grain color of Jigu 31; **(C–E)**, brown hull color of selected lines; **(F, G)**, yellow hull and grain colors of Yugu 5; **(H–J)**, yellow hull color of selected lines.

Yugu 5 and Jigu 31 displayed yellow and brown hull colors, respectively, in all environments either in the higher-latitude site (Cangzhou, Hebei province) in summer seasons, or in the lower-latitude site (Sanya, Hainan province) in winter seasons in 2016 and 2017 (**Figures 1A, F**). It was able to categorize unambiguously hull colors of the RILs as yellow and brown regardless of environments, but there was slight difference in the color intensity among lines in each category (**Figures 1C–E, H–J**). Hull colors of all the progeny lines were generally consistent with slight variation in color intensity in different locations or years examined, demonstrating that environment has little effect on the hull color performance. The color of hulls was not associated with colors of hulless grains, as the later was always yellow for both Yugu 5 and Jigu 31 (**Figures 1B, G**). The RIL population segregated for 182 yellow and 101 brown hull colored lines, which agrees with a ratio of 2 (yellow): 1 (brown) (χ^2 =^ 0.6047, *P*=0.4368) for segregation of multiple loci, rather than in a 1:1 segregation ratio for single locus.

### BSR-seq-based identification of hull color-associated genomic regions

**Figure 2:**
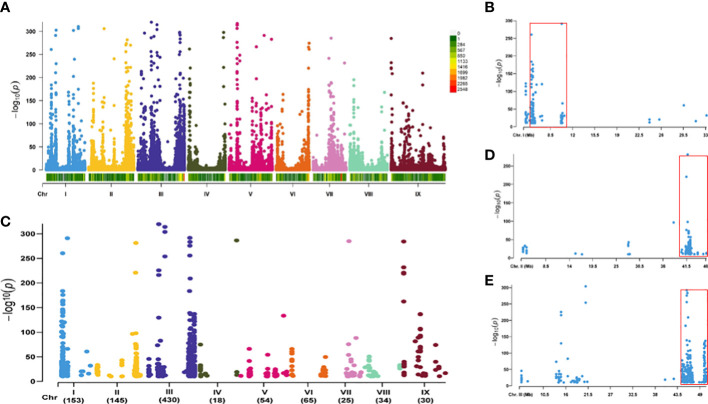
SNPs identified by the BSR-Seq analysis based on the RILs of Yugu 5 × Jigu 31 cross with yellow and brown hull colors. **(A)** distribution of all SNPs generated by RNA sequencing of the two bulks for the RNA samples of the RILs yellow and brown hull colors (Bulk-Y and Bulk-B, respectively) on different foxtail millet chromosomes. **(B)** distribution of SNP variants between Bulk-Y and Bulk-B potentially associated with hull color produced by BSR-Seq analysis on different foxtail millet chromosomes. **(C–E)**, SNP variants on chromosomes 1, 2, and 3. The significantly enriched genomic intervals of the candidate SNPs for hull color are marked with red boxes.

The statistical parameters generated by RNA sequencing for the yellow- and brown-hulled lines, Bulk-Y and Bulk-B, are shown in **Table 1**. Most reads for both samples, 93.0% for Bulk-Y and 95.5% for Bulk-B, were mapped to the foxtail millet reference genome. A total of 117,260 SNP variants were identified from the mapped reads by the Strelka software with default parameters, which were scattered on the nine foxtail millet chromosomes (**Figure 2A**). Among them, 956 variants between Bulk-Y and Bulk-B were potentially trait-associated (*P*-value <1e-10 and AFD >0.8), which were anchored on all foxtail millet chromosomes (**Figure 2B**). Chromosomes 1, 2, and 3 harbored the most abundant number of candidate trait-associated SNP variants of 153, 145, and 430, respectively, which appear to be the critical chromosomes associated with hull color performance. Chromosomes 4 to 9 had a smaller number of SNP variants from 18 to 65 and are unlikely associated with the hull color trait. The SNP clusters on chromosomes 1, 2, and 3 were located in 6.13 Mb (4,570,517-10,698,955 bp, 146 SNP loci) (**Figure 2C**), 5.87 Mb (40,301,380-46,168,003 bp, 122 SNP loci) (**Figure 2D**), and 6.06 Mb (44,469,860-50,532,757 bp, 362 SNP loci) (**Figure 2E**) genomic intervals, respectively, indicating their potential association with the hull color trait. *P* = 0.036

**Table 1:** Statistical parameters of the BSR-Seq analysis for the yellow hulled lines (Bulk-Y) and brown-hulled lines (Bulk-B).

RNA sample	No. of raw read pairs	high-quality read pairs	Mapped read pairs^a^	
			No. of reads	%
Bulk-Y	26,573,357	26,572,236	24,699,160	93.0
Bulk-B	30,129,826	30,128,582	28,772,504	95.5

a The read pairs that are mapped to the Setaria italica reference genome (http://plants.ensembl.org/Setaria_italica/Info/Annotation/#assembly).

### Linkage map-based identification of QTL for hull color

**Figure 3:**
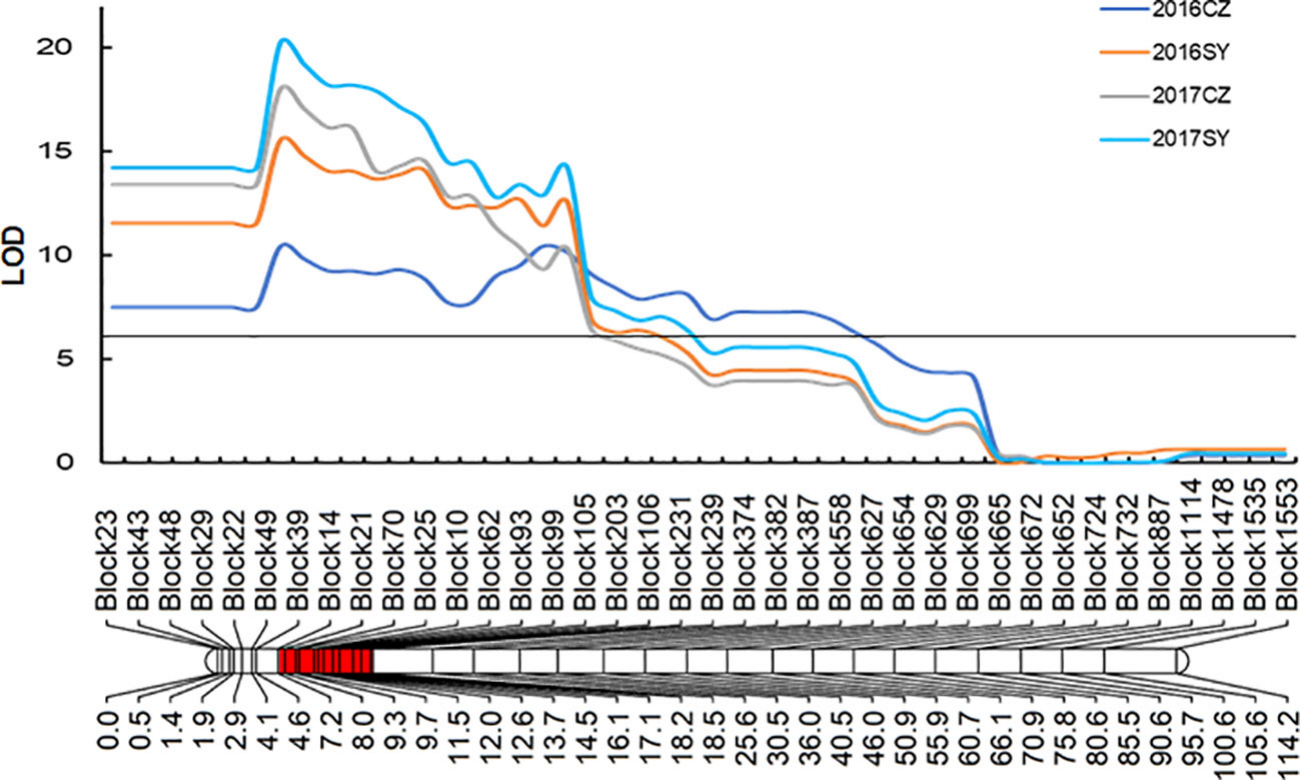
Genetic linkage of *QHC-czas1* on chromosome 1 conferring brown hull color of Jigu 31. CZ, Cangzhou site; SY, Sanya site.

A genetic linkage map was previously constructed by means of re-sequencing 147 RILs from cross Yugu × Jigu 31, which spanned a map distance of 1806.77 cM (Tian et al., 2021). A QTL designated *QHC-czas1.1*, was detected between bin markers Block43 and Block697 in all environments (**Table 2** and **Figure 3**). The LOD values of this QTL ranged from 10.5 to 20.3, and the phenotypic variations explained were in the range of 28.0% to 47.0%. This genetic interval corresponded a genomic region of 18.14 Mb (2,236,038-20,380,190 bp) and overlapped with the genomic region on chromosome 1 (4,570,517-10,698,955 bp) for hull color identified by the BSR-Seq analysis. Because Jigu 31 contributed to this QTL, it controls brown hull color.

**Table 2:** QTL conferring brown hull color identified with the recombinant inbred line population of Yugu 5 × Jigu 31 in four field trials.

Trial^a^	Marker interval	Physical interval	Logarithm of odds	Phenotypic variation explained (%)	Additive
2016CZ	Block43-Block689	2,236,038-19,676,921	10.5	28.0	0.2740
2016SY	Block43-Block697	2,236,038-20,380,190	15.6	38.6	0.3051
2017CZ	Block43-Block625	2,236,038-12,448,008	18.1	43.2	0.3322
2017SY	Block43-Block687	2,236,038-19,420,325	20.4	47.0	0.3452

a CZ, Cangzhou; Hebei province; SY, Sanya; Hainan province.

### Annotation and expression of candidate genes

Base on the results of BSR-Seq analysis, three candidate genomic regions on chromosomes 1, 2, and 3 were associated with the hull color. These genomic regions contained various number of genes related to anthocyanin synthesis, including Myc-type basic helix-loop-helix (bHLH) domain genes, SANT/Myb domain genes, WD40 repeat, helix-loop-helix DNA-binding domain superfamily and WD40/YVTN repeat-like-containing domain superfamily (**Table 3**). The candidate genomic region on chromosome 1 (6.13 Mb, 4,570,517-10,698,955 bp) overlapped with the physical interval (5,486,321-5,495,814 bp) of *QHC-czas1.1* detected by re-sequencing of the RIL population (**Table 1**). This genomic region included 18 genes associated with anthocyanin synthesis out of 574 annotated genes. Gene *Si1g06530* was commonly detected in the genomic regions identified by BSR-Seq and the map-based analyses.

**Table 3:** Annotated genes in the genomic regions associated with hull color identified by the BSR-Seq analysis.

Gene stable ID	Gene start (bp)	Gene end (bp)	Annotation
Chromosome 1			
*Si1g04700*	4,926,459	4,928,548	Myb/SANT-like domain
*Si1g05980*	5,001,092	5,004,976	SANT/Myb domain
*Si1g06490*	5,474,789	5,475,402	Myb/SANT-like domain
*Si1g06530*	5,499,541	5,501,195	SANT/Myb domain
*Si1g06860*	5,791,855	5,796,147	SANT/Myb domain
*Si1g07130*	6,069,305	6,069,838	Helix-loop-helix DNA-binding domain superfamily
*Si1g07580*	6,423,556	6,425,841	SANT/Myb domain
*Si1g08830*	7,438,180	7,444,525	WD40 repeat
*Si1g09680*	8,156,612	8,159,790	SANT/Myb domain
*Si1g09850*	8,277,128	8,287,793	WD40 repeat
*Si1g10660*	8,928,257	8,940,446	WD40 repeat
*Si1g10910*	9,130,853	9,131,784	Myc-type, basic helix-loop-helix (bHLH) domain
*Si1g11400*	9,633,197	9,635,600	Myc-type, basic helix-loop-helix (bHLH) domain
*Si1g12040*	10,216,744	10,218,257	SANT/Myb domain
*Si1g12060*	10,226,010	10,227,062	WD40 repeat
*Si1g12170*	10,278,397	10,281,314	Myc-type, basic helix-loop-helix (bHLH) domain
*Si1g12350*	10,409,810	10,411,875	SANT/Myb domain
*Si1g12510*	10,609,344	10,610,474	Myb/SANT-like domain
Chromosome 2			
*Si2g32330*	40,974,148	40,975,501	SANT/Myb domain
*Si2g32790*	41,316,187	41,320,406	WD40 repeat
*Si2g34400*	42,480,225	42,483,305	Myc-type, basic helix-loop-helix (bHLH) domain
*Si2g34740*	42,730,416	42,732,136	Myc-type, basic helix-loop-helix (bHLH) domain
*Si2g35120*	43,077,106	43,079,325	Myc-type, basic helix-loop-helix (bHLH) domain
*Si2g35280*	43,178,814	43,180,348	SANT/Myb domain
*Si2g36170*	43,835,381	43,838,038	WD40 repeat
*Si2g37310*	44,663,976	44,665,285	Myc-type, basic helix-loop-helix (bHLH) domain
*Si2g37880*	45,033,715	45,037,272	WD40 repeat
*Si2g39450*	46,092,543	46,096,478	WD40 repeat
Chromosome 3			
*Si3g34550*	45,040,962	45,043,436	SANT/Myb domain
*Si3g34880*	45,481,965	45,482,870	Myc-type, basic helix-loop-helix (bHLH) domain
*Si3g35440*	46,120,623	46,125,823	WD40/YVTN repeat-like-containing domain superfamily
*Si3g35530*	46,434,249	46,441,702	SANT/Myb domain
*Si3g36330*	47,403,180	47,405,428	SANT/Myb domain
*Si3g36380*	47,440,375	47,441,867	Myc-type, basic helix-loop-helix (bHLH) domain
*Si3g36530*	47,669,444	47,671,374	SANT/Myb domain
*Si3g36570*	47,707,799	47,709,565	WD40 repeat
*Si3g36750*	47,813,301	47,817,514	WD40 repeat
*Si3g36930*	47,954,102	47,957,355	Myc-type, basic helix-loop-helix (bHLH) domain
*Si3g36940*	47,960,405	47,961,703	Myc-type, basic helix-loop-helix (bHLH) domain
*Si3g36970*	47,997,132	48,000,399	Myc-type, basic helix-loop-helix (bHLH) domain
*Si3g37620*	48,675,920	48,682,059	WD40 repeat
*Si3g37640*	48,686,318	48,690,089	Myc-type, basic helix-loop-helix (bHLH) domain
*Si3g38390*	49,341,776	49,344,227	WD40 repeat
*Si3g39300*	50,139,658	50,143,141	Myc-type, basic helix-loop-helix (bHLH) domain
*Si3g36950*	47,979,765	47,980,422	Myc-type, basic helix-loop-helix (bHLH) domain
*Si3g36980*	48,031,195	48,034,194	Myc-type, basic helix-loop-helix (bHLH) domain

The SNPs that were associated with hull color on chromosome 2 were clustered in a 5.87 Mb (40,301,380-46,168,003 bp) genomic interval, which composes 886 annotated genes including 10 genes for anthocyanin synthesis. A 6.06 Mb (44,469,860-50,532,757 bp) genomic interval consisted of the SNP cluster on chromosome 3. It consisted of 651 annotated genes, including 18 genes for anthocyanin synthesis (**Table 3**). The genes associated with anthocyanin synthesis on these chromosomes include Myc-type basic helix-loop-helix (bHLH) domain genes (13 genes), SANT/Myb domain genes (6 genes), WD40 repeat (8 genes), and WD40/YVTN repeat-like-containing domain superfamily (one gene).

**Figure 4:**
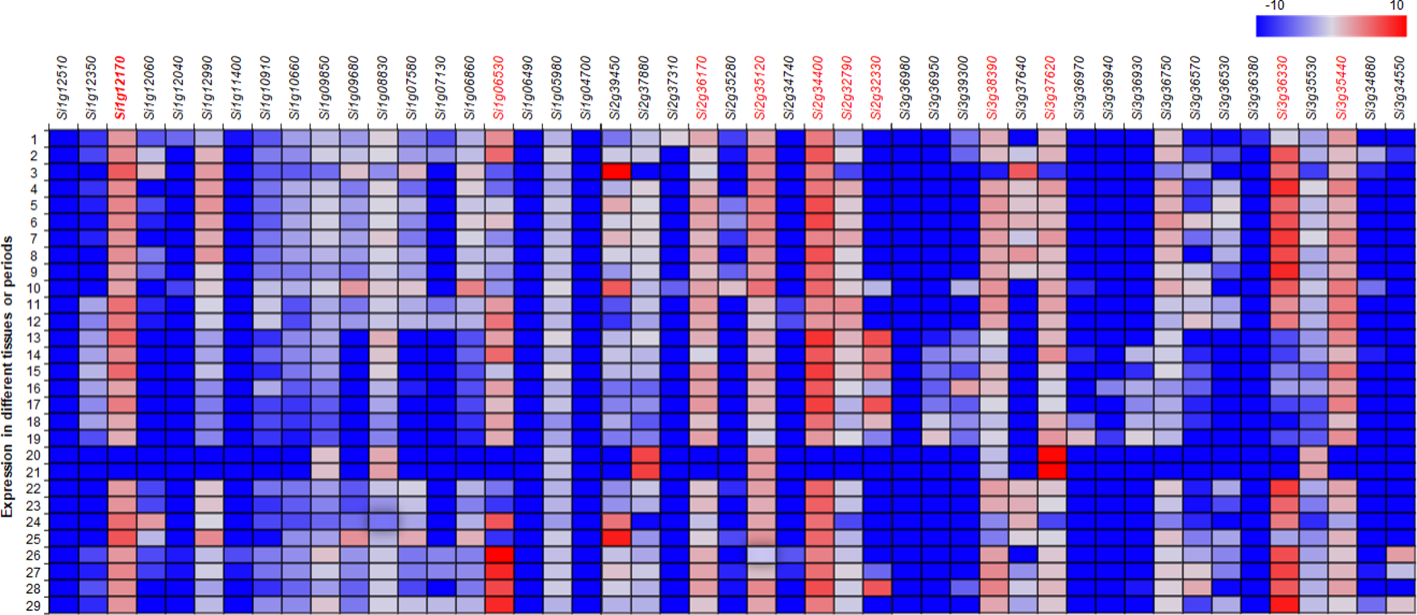
Heatmap for the expression of the annotated genes related to anthocyanin biosynthesis in the candidate genomic regions in 29 datasets of expression in foxtail millet. The datasets were extracted from MDSi: Multi-omics Database for *Setaria italica* (http://foxtail-millet.biocloud.net/). Genes expressed in developing seeds and panicles are indicated by red fonts. 1, JG21_Germinated-seeds_3-days; 2, JG21_Plant_one-tip-two-leaf; 3, JG21_Leaf-top-2-3_2-days-after-heading; 4, JG21_Neck-panicle-internodes_Filling-stage; 5, JG21_Flag-leaf_filling-stage; 6, JG21_Flag-leaf-sheath_filling-stage; 7, JG21_Stem-top-second_Filling-stage; 8, JG21_Leaf-top-foruth_filling-stage; 9, JG21_Leaf-sheath-top-foruth_filling-stage; 10, JG21_Root_Filling-stage; 11, JG21_Panicle_Primary-panicle-branch-differentiation-stage; 12, JG21_Panicle_Third-panicle-branch-differentiation-stage; 13, JG21_Immature-spikelet_S2; 14, JG21_Immature-spikelet_S4; 15, JG21_Immature-seed_S1; 16, JG21_Immature-seed_S2; 17, JG21_Immature-seed_S3; 18, JG21_Immature-seed_S4; 19, JG21_Immature-seed_S5; 20, JG21_Seed_30-days-after-maturation; 21, JG21_Seed_60-days-after-maturation; 22, JG21_Leaf-veins_S3; 23, JG21_Mesophyll_S3; 24, Xiaomi_Leaf_3-weeks-plant; 25, Xiaomi_Leaf-top-second_Boot-stage; 26, Xiaomi_Panicle_2-days-after-heading; 27, Xiaomi_Panicle_Pollination-stage; 28, Xiaomi_Panicle_Filling-stage; and 29, Xiaomi_Stem_Filling-stage.

The transcriptional patterns of the genes related to anthocyanin synthesis were analyzed with a web-based database containing 29 sets of expressions for different growth stages of foxtail millet (http://foxtail-millet.biocloud.net/page/tools/expressionVisualization). Genes *Si1g06530* (SANT/Myb domain) and *Si1g12170* (bHLH) on chromosome 1, *Si2g32330* (SANT/Myb domain), *Si2g32790* (WD40 repeat), *Si2g34400* (bHLH), *Si2g35120* (bHLH), and *Si2g36170* (WD40 repeat) on chromosome 2, and *Si3g34550* (SANT/Myb domain), *Si3g36330* (SANT/Myb domain), *Si3g37620* (WD40 repeat), and *Si3g38390* (WD40 repeat) on chromosome 3 were expressed in developing panicles and seeds at different developmental stages (**Figure 4**).

## Discussion

Hull color is an obvious phenotypic trait that can be visualized easily. Segregation of yellow and brown hull colors with different color intensities in the RIL population derived from Yugu 5 × Jigu 31 indicates that multiple genetic loci are involved in controlling hull color. We identified three genomic regions on chromosomes 1, 2, and 3 for brown hull color of Jigu 31 by means of the BSR-Seq analysis. We further identified a QTL, *QHC-czas1.1*, on chromosome 1 in Jigu 31 using a genetic map constructed with re-sequencing of the RIL population. This QTL plays a major role in governing hull color as the phenotypic variations explained was as high as 28.0%-47.0%.

Foxtail millet accumulates pigments on husks, making seeds appear different colors (Wang et al., 2016). Jia et al. (2013) carried out a GWAS project and identified 11 SNP variants that are associated with hull color on chromosomes 1, 6, 7, and 9 of foxtail millet. Three of them were detected in genomic loci at 5,480,719, 5,467,847, and 5,476,983 bp on chromosome 1. A QTL, *Sihc1*, on chromosome 6 was reported to confer green hull color (Wang et al., 2017). Li et al. (2015) identified a single locus *SeC-1* conferring hull color on chromosome 7 using a residue heterozygous line differing in red and black hull colors. These results also provide evidence that hull color of foxtail millet is controlled by different genetic loci.

Pigmentation in plant tissues results from anthocyanin biosynthesis, which involves structural and regulatory genes. Several classes of transcription factors, such as MYB, bHLH, and WD40, are known to regulate anthocyanin synthesis in various plant species, such as *Arabidopsis thaliana* L., maize (*Zea mays* L.), and tomato (*Solanum lycopersicum* L.) (Broun, 2005; Koes et al., 2005; Petroni and Tonelli, 2011). In this study, we identified a number of genes for the classes of transcription factor genes containing bHLH, SANT/Myb, and WD40 domains in the genomic regions on chromosomes 1, 2, and 3 that are associated with hull color. Some of them are highly expressed in developing panicles or seeds. In particular, *Si1g06530* encoding a SANT/Myb domain protein was commonly detected by the analyses of BSR-Seq and the genetic linkage map. It is preferentially expressed in several data sets in the developmental stages of panicle differentiation, immature-spikelets and immature-seeds. This gene warrants further verification as a candidate gene regulating hull color by genomic editing and transgenic approaches.

Pigmentation also occurs in other millet tissues, such as leaf blade and sheath, pulvinus, anther, pericarp, aleurone, and grain, which involves different genetic mechanisms for regulating the accumulation of anthocyanin. The accumulation of pigmentations in different tissues may not be necessarily related. For example, the grain color of Yugu 5 and Jigu 31 is yellow despite that these cultivars differ in their hull color. Pigmentation in millet tissues is beyond as an apparent trait, it may be associated with functional properties that benefit humans. Therefore, many studies have been carried out to understand the genetic controls of pigmentation in foxtail millet. Although the genetic mechanisms underpinning biosynthesis of anthocyanin in other plant species are well characterized (Koes et al., 2005; Petroni and Tonelli, 2011), the regulatory mechanism of pigmentation in foxtail millet tissues is rarely studied until a recent report by Bai et al. (2020). That study mapped a locus *PPLS1* on chromosome 7 conferring the color in pulvinus and leaf sheath in foxtail millet. *PPLS1* proved to interact with a MYB transcription factor for regulating the expression of anthocyanin.

A rice gene *BBH*/*Lsi1* on chromosome 2 regulates black-brown hull of rice by reducing silicon deposition and accumulating flavonoid (Yang et al., 2018). Sun et al. (2018) proposed a *C-S-A* gene model for pigmentation of rice hull. *C1*, a color-producing gene encoding a R2R3-MYB transcription factor, interacts with *S1*, encoding a bHLH protein, and activates expression of *A1*, encoding a dihydroflavonol reductase. As the reference genome sequence of foxtail millet is available, it may not be difficult to develop more molecular markers to finely map and cloning of the candidate genes and disclose the regulatory mechanism underlying hull color trait. The availability of genome sequences for a large number of foxtail millet cultivars and breeding lines with clear hull color performances and multi-omics approaches will facilitate this process (Jia et al., 2013; Li et al., 2022). The identification of genomic regions associated with anthocyanin biosynthesis, in particular highly expressed gene *Si1g06530* in panicle, can serve as a good start for studying regulatory mechanism of hull color in foxtail millet.

In summary, we identified a major QTL for brown hull color of foxtail millet cultivar Jigu 31 on chromosome 1 using a genetic link map constructed with re-sequencing a RIL population of cross Yugu 5 × Jigu 31. Three genomic regions associated with hull color were identified on chromosome 1, 2, and 3 by BSR-Seq analysis. Forty-seven genes related for anthocyanin biosynthesis were observed in these genomic regions. Some of them were highly expressed in developing panicles and seeds of foxtail millet. Gene *Si1g06530* encoding a SANT/Myb domain protein on chromosome 1 was present in the overlapped genomic region commonly detected by the genetic linkage map and BSR-Seq analyses, which can be a candidate gene for the brown hull color of Jigu 31.

## Data availability statement

The data presented in the study are deposited in the National Genomics Data Center (https://ngdc.cncb.ac.cn/;NGDC) repository, accession number CRA008186.

## Authors contributions

HL and BT conceived and designed the study. BT developed the population. BT, LXZ, YL, WP, and LLZ conducted the field experiments. JH performed sequencing data analysis. HL wrote the manuscript. All authors contributed to the article and approved the submitted version.

## Funding

The financial support provided by the Ministry of Agriculture of P.R. China (CARS-06-13.5-B1) is gratefully acknowledged.

## Conflict of interest

The authors declare that the research was conducted in the absence of any commercial or financial relationships that could be construed as a potential conflict of interest.

## Publisher’s note

All claims expressed in this article are solely those of the authors and do not necessarily represent those of their affiliated organizations, or those of the publisher, the editors and the reviewers. Any product that may be evaluated in this article, or claim that may be made by its manufacturer, is not guaranteed or endorsed by the publisher.
